# Epidemiology and the economic burden of traumatic fractures in China: A population-based study

**DOI:** 10.3389/fendo.2023.1104202

**Published:** 2023-01-24

**Authors:** Bo-xuan Huang, Yan-hua Wang, Hai-bo Wang, Chu Wang, Fei-fei Jin, Jing Li, Lan-xia Gan, Ying Shi, Bao-guo Jiang, Dian-ying Zhang

**Affiliations:** ^1^ Department of Orthopedics and Trauma, Peking University People’s Hospital, Beijing, China; ^2^ Key Laboratory of Trauma and Neural Regeneration (Peking University), Ministry of Education, Beijing, China; ^3^ National Center for Trauma Medicine, Beijing, China; ^4^ Clinical Trial Unit, First Affiliated Hospital of Sun Yat-Sen University, Guangzhou, China; ^5^ Trauma Medicine Center, Peking University People’s Hospital, Beijing, China; ^6^ China Standard Medical Information Research Center, Shenzhen, China

**Keywords:** traumatic fractures, population-based study, epidemiology, admission rate, in-hospital mortality, economic burden

## Abstract

**Objectives:**

National data on the admission rate, distribution, in-hospital mortality, and economic burden of traumatic fractures in China is unclear. We aimed to conduct a cross-sectional population-based study to determine such above data at the national level in China.

**Methods:**

A national administrative database was used to review all traumatic fracture hospitalizations in China during 2020, from which a total of 2,025,169 inpatients with traumatic fractures was retrieved. Admission rates and in-hospital mortality rates stratified by age, sex, and region were calculated. The causes of traumatic fracture and economic burden were described.

**Results:**

The admission rate of traumatic fractures of all China population in 2020 was 1.437‰. The admission rate increased with age and varied with genders and causes of injuries. Falls are the leading cause of traumatic fracture hospitalization, followed by road traffic injuries. The most common diagnoses were femoral neck fractures, with a number of 138,377. The in-hospital mortality was 1.209‰. Road traffic injuries led to the highest in-hospital mortality. The median length of stay was 10 days, with the median hospitalization cost of ¥20,900 (about $3,056).

**Conclusion:**

Traumatic fractures are concerning conditions with a high admission rate and in-hospital mortality in China, which are mainly caused by falls and road traffic injuries. The government should implement more public health policies to enhance the health of the elderly and improve transportation safety to prevent traumatic fractures.

## 1 Introduction

Trauma is a major cause of mortality and disability worldwide. In China, trauma is the fifth leading cause of death among the whole population and the first leading cause of death among young adults (1). Fractures are often secondary to traumatic injuries and severely affect patients’ quality of life. The GBD 2019 study estimated there were 2296.2 new incident cases of traumatic fractures and 319.0 YLDs per 100,000 population worldwide in 2019 (2). Since traumatic fractures impose a huge burden on medical services, it is essential to study on the epidemiology of fractures. However, previous studies conducted in high-income countries showed a large variation in the incidence and mortality rate of traumatic fracture (3–13). Also, these findings did not apply to developing countries like China due to socioeconomic development, culture, and lifestyle differences.

In recent years, more and more Chinese researchers focused on the epidemiological characteristics of traumatic fractures in China (14–17), but national data on the epidemiology and the economic burden of traumatic fracture hospitalization in China is still scarce. China is a huge developing country with a vast territory and a large population, and there is an imbalance in economic development, cultural customs, and health services among different regions, which makes conducting nationwide epidemiology studies a hard task. Using administrative databases to obtain nation-specific data may be a feasible way (18). Hence, we utilized a nationwide inpatient discharge database to conduct a cross-sectional study and attempted to describe the epidemiology characteristics and economic burden of traumatic fracture in China, so as to provide a scientific basis for the formulation of health policies.

## 2 Methods

### 2.1 Data source

The Hospital Quality Monitoring System (HQMS) of the National Health Commission covers all public tertiary hospitals in China (excluding Taiwan, Hong Kong, and Macau) and collects the medical record homepage of all patients admitted into these hospitals (19–21). From the HQMS database, 4,290,877 inpatients with trauma and 2,025,169 inpatients with traumatic fracture were reviewed between January 1^st^ and December 31^st^ 2020. 1947 tertiary public hospitals were included in this study, accounting for 97.2% of all tertiary public hospitals nationwide.

### 2.2 Definition of inpatient with trauma and traumatic fracture

In ICD-10 coding system, S00-S99 and T00-T35 are diagnoses for traumas. Inpatients with a primary diagnosis of trauma were defined as inpatients with a trauma. From these diagnoses, the ones containing the word “fracture” were selected, while diagnoses such as “skull fracture”, “old fracture”, “sequelae of fracture”, “cartilage fracture” and “internal fixation of fracture” were excluded, and the remaining 436 diagnoses were defined as diagnoses for traumatic fractures, as is shown in online **Supplementary Table 1**. Inpatients with a primary diagnosis of traumatic fracture were defined as inpatients with a traumatic fracture.

### 2.3 Data collection

Gender, age, causes of injury, primary diagnosis, procedure, hospitalization costs, length of stay (LOS), and outcome were retrieved from inpatients’ medical records. Gender was male or female. Age was divided into a total of 18 groups for every 5 years. The causes of injury were road traffic injury, fall, cutting/perforating/firearm injury, blunt/impact injury, animal attack, explosion/burn injury, mechanical injury, and other reasons. According to the location where the fracture occurred, patients were grouped into one of six regions: North China, Northeast China, East China, South Central China, Southwest China, and Northwest China. Hospitalization cost was the total medical costs of inpatients in CNY (¥) as well as USD ($).

### 2.4 Admission rate and in-hospital mortality

The admission rate of traumatic fractures was defined as the ratio of the number of inpatients with traumatic fractures to the total national population of China in 2020. Standard population data were obtained from the seventh census conducted by the National Bureau of Statistics of China in 2020 (22). The same method was used to calculate the admission rates stratified by ages, genders, and regions. In-hospital mortality was defined as the ratio of the number of death cases to the total number of inpatients.

### 2.5 Statistical methods

For categorical variables, frequencies and proportions (%) were used; for continuous variables, data were presented as median with interquartile range (IQR). We did not report P values because the large sample data of HQMS might lead to statistically significant P values, but they might not be clinically significant due to the small absolute differences. Statistical analyses were conducted using SAS 9.4 (SAS Institute Inc., USA). The choropleth maps of the admission rate, in-hospital mortality, hospitalization cost, length of stay were made using Excel 2010 (Microsoft Co., Ltd, USA) to describe the distribution characteristics.

## 3 Result

### 3.1 Gender and age

**Figure 1:**
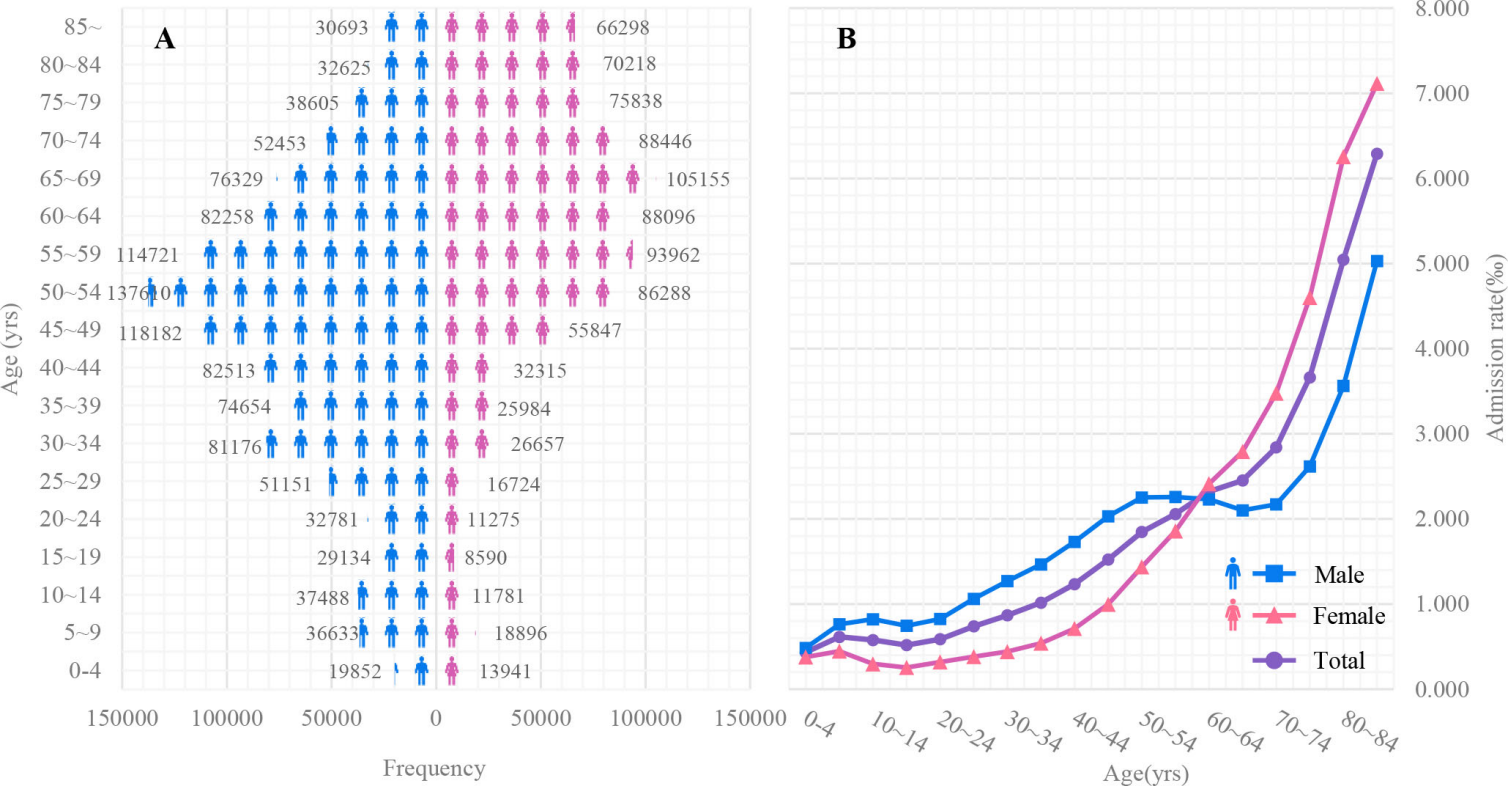
The frequency **(A)** and admission rate **(B)** for male and female inpatients with traumatic fractures among different age groups in China in 2020.

The overview of the total of 2025169 inpatients with traumatic fractures in China in 2020 are summarized in **Table 1**. The admission rate of traumatic fractures in China in 2020 was 1.437 per 1,000, and 1.565 per 1000 in males and 1.302 per 1000 in females, respectively. Males had more fractures than females at 0-59 years of age, and females had more fractures over 60 years of age. The admission rate of traumatic fractures showed an upward trend with age. Before the sixth decade of life, the admission rate was always higher in men than in women, while after that age, the opposite happened (see **Figure 1**). The age and gender-stratified epidemiology characteristics is presented in online **Supplementary Table 2**.

**Table 1:** A overview of the characteristics and the economic burden of inpatients with traumatic fractures in China in 2020.

Categories	Total number, N (%)	Crude incidence, ‰	Death number, N (%)	In-hospital mortality, ‰	Length of stay, Median (IQR), Days	Hospitalization Cost, Median(IQR), ¥10,000.00
**Total**	2025169 (100.00)	1.437	2448 (100.00)	1.209	10.0 (6.0 to 16.0)	2.09 (0.77 to 3.77)
Gender						
Male	1128858 (55.74)	1.565	1466 (59.89)	1.299	10.0 (6.0 to 17.0)	1.92 (0.73 to 3.67)
Female	896311 (44.26)	1.302	982 (40.11)	1.096	10.0 (6.0 to 16.0)	2.29 (0.86 to 3.86)
Age						
0~4	33793 (1.67)	0.434	2 (0.08)	0.059	5.0 (3.0 to 8.0)	1.00 (0.54 to 1.51)
5~9	55529 (2.74)	0.615	3 (0.12)	0.054	6.0 (4.0 to 9.0)	1.12 (0.62 to 1.70)
10~14	49269 (2.43)	0.578	15 (0.61)	0.304	7.0 (4.0 to 11.0)	1.30 (0.58 to 2.20)
15~19	37724 (1.86)	0.519	21 (0.86)	0.557	9.0 (5.0 to 15.0)	1.69 (0.67 to 3.07)
20~24	44056 (2.18)	0.588	28 (1.14)	0.636	9.0 (5.0 to 16.0)	1.84 (0.69 to 3.43)
25~29	67875 (3.35)	0.739	43 (1.76)	0.634	9.0 (5.0 to 16.0)	1.86 (0.70 to 3.46)
30~34	107833 (5.32)	0.869	63 (2.57)	0.584	10.0 (6.0 to 16.0)	1.91 (0.71 to 3.55)
35~39	100638 (4.97)	1.016	49 (2.00)	0.487	10.0 (6.0 to 17.0)	1.98 (0.74 to 3.65)
40~44	114828 (5.67)	1.235	62 (2.53)	0.540	11.0 (6.0 to 18.0)	1.95 (0.71 to 3.66)
45~49	174029 (8.59)	1.524	103 (4.21)	0.592	11.0 (7.0 to 18.0)	1.90 (0.69 to 3.64)
50~54	223898 (11.06)	1.848	159 (6.50)	0.710	11.0 (7.0 to 18.0)	1.93 (0.70 to 3.66)
55~59	208683 (10.30)	2.058	159 (6.50)	0.762	11.0 (6.0 to 17.0)	2.08 (0.76 to 3.77)
60~64	170354 (8.41)	2.321	168 (6.86)	0.986	10.0 (6.0 to 17.0)	2.32 (0.88 to 4.00)
65~69	181484 (8.96)	2.452	209 (8.54)	1.152	10.0 (6.0 to 16.0)	2.43 (0.94 to 4.06)
70~74	140899 (6.96)	2.841	196 (8.01)	1.391	10.0 (6.0 to 16.0)	2.58 (1.00 to 4.13)
75~79	114443 (5.65)	3.663	270 (11.03)	2.359	10.0 (6.0 to 16.0)	2.74 (1.07 to 4.18)
80~84	102843 (5.08)	5.046	335 (13.68)	3.257	10.0 (6.0 to 16.0)	2.94 (1.16 to 4.31)
85~	96991 (4.79)	6.291	563 (23.00)	5.805	10.0 (6.0 to 16.0)	3.03 (1.06 to 4.47)
Region						
North China	233961 (11.55)	1.382	357 (14.58)	1.526	10.0 (6.0 to 16.0)	2.80 (1.27 to 4.69)
Northeast China	138565 (6.84)	1.407	324 (13.24)	2.338	10.0 (6.0 to 18.0)	2.69 (1.11 to 4.46)
East China	717079 (35.41)	1.693	716 (29.25)	0.998	9.0 (6.0 to 15.0)	2.21 (0.92 to 3.80)
Central South China	461386 (22.78)	1.126	553 (22.59)	1.199	11.0 (6.0 to 18.0)	1.81 (0.63 to 3.49)
Southwest China	319969 (15.80)	1.560	337 (13.77)	1.053	11.0 (6.0 to 18.0)	1.50 (0.52 to 3.11)
Northwest China	154209 (7.61)	1.488	161 (6.58)	1.044	10.0 (6.0 to 16.0)	2.01 (0.76 to 3.57)
Cause						
Road traffic injury	439930 (23.46)	NA	778 (36.34)	1.768	13.0 (7.0 to 21.0)	1.96 (0.71 to 3.87)
Fall	1119653 (59.70)	NA	1195 (55.82)	1.067	10.0 (6.0 to 15.0)	2.22 (0.85 to 3.75)
Cutting/Perforating/Firearm injury	5697 (0.30)	NA	4 (0.19)	0.702	6.0 (4.0 to 11.0)	0.96 (0.58 to 1.68)
Blunt/Impact injury	81892 (4.37)	NA	56 (2.62)	0.684	10.0 (6.0 to 17.0)	1.19 (0.52 to 2.78)
Animal attack	1410 (0.08)	NA	0 (0.00)	0.000	10.0 (6.0 to 17.0)	1.55 (0.61 to 3.32)
Explosion/Burn injury	1810 (0.10)	NA	3 (0.14)	1.657	11.0 (6.0 to 21.0)	1.44 (0.72 to 3.46)
Mechanical injury	92616 (4.94)	NA	28 (1.31)	0.302	8.0 (5.0 to 15.0)	1.15 (0.65 to 2.30)
Other reasons	132362 (7.06)	NA	77 (3.60)	0.582	8.0 (5.0 to 14.0)	2.25 (0.91 to 3.83)

IQR, interquartile range; NA, not applicable.

### 3.2 Regions

**Figure 2:**
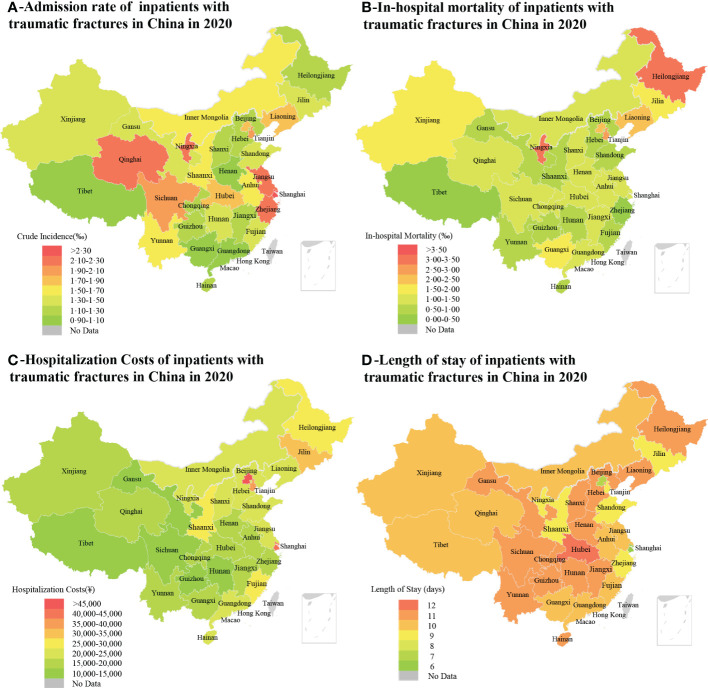
The choropleth maps of admission rate **(A)**, in-hospital mortality **(B)**, hospitalization cost **(C)**, and length of stay **(D)** of inpatients with traumatic fractures for 31 provinces, municipalities and autonomous regions in China in 2020. The study was conducted in mainland China, and the data of Hong Kong, Macao and Taiwan were not displayed (grey color).

The number of traumatic fractures was the highest in East China, because the number of tertiary public hospitals and population in this region were higher than those in other regions; similarly, the number of cases in Northeast China was the lowest, see **Table 1**. As shown in online **Supplementary Table 3**, the admission rate of traumatic fracture in each province ranged from 0.905 to 2.769 per 1000, and the provinces with higher incidence were mainly in East China, North China, and Northwest China (see **Figure 2**).

### 3.3 Causes of traumatic fractures

Falls were the most common cause of traumatic fractures, followed by road traffic injuries, which together accounted for more than 80% of cases. The number of traumatic fractures caused by animal attacks was lowest. Males were prone to be injured for all kinds of reasons. The numbers of inpatients with traumatic fractures caused by explosion/burn injuries, mechanical injuries, and blunt/impact injuries were much higher in males than in females (>4:1).

**Figure 3:**
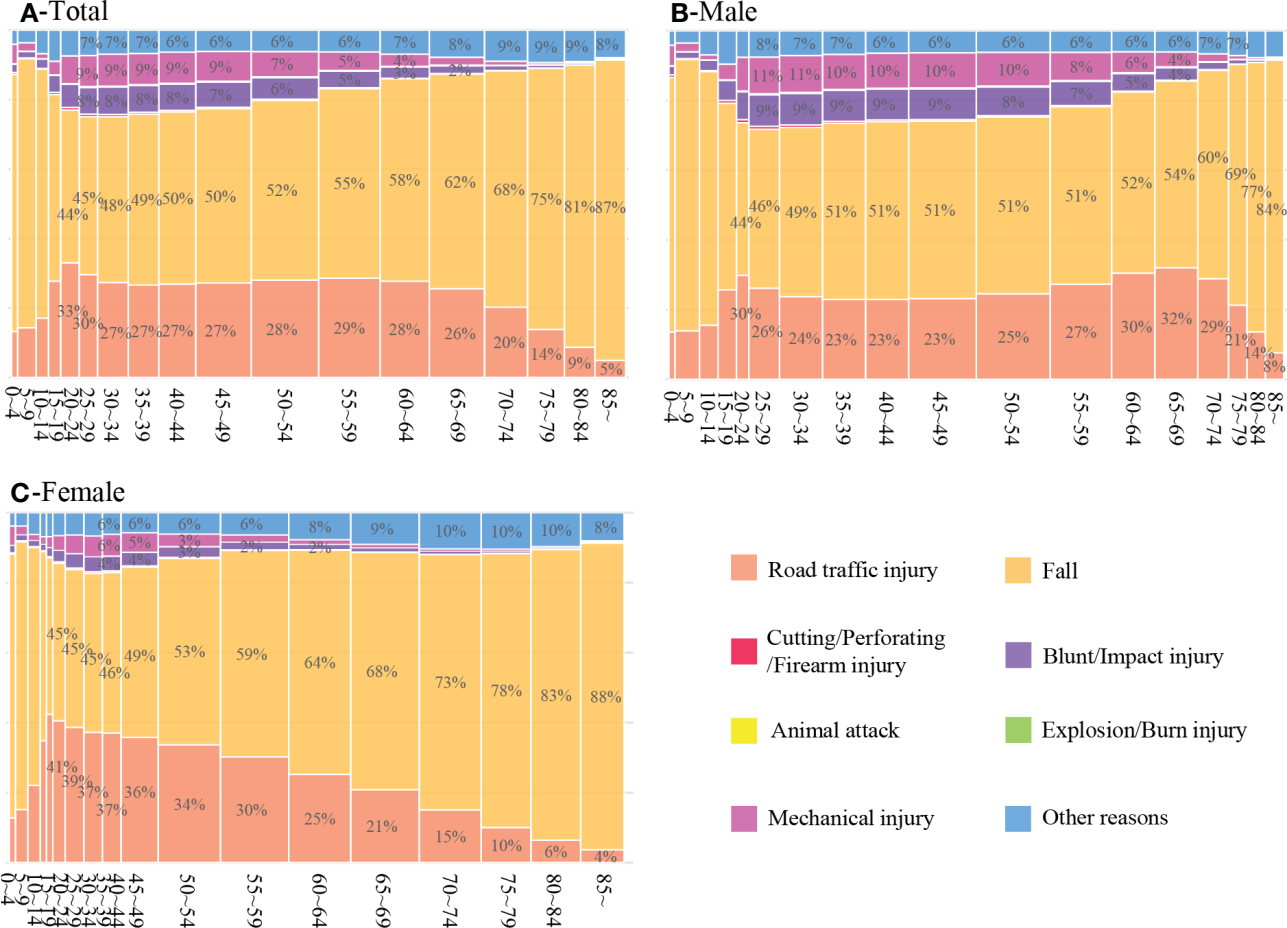
Mosaic plots of the distribution of causes of traumatic fracture in total **(A)**, male **(B)** and female **(C)** inpatients of different age groups. (Some percentage cannot be displayed because it is relatively small).

**Table 2** shows the distribution of causes of fracture stratified by age and gender. At all ages, falls and road traffic injuries were the top two causes of injuries. Traumatic fractures due to falls took a higher proportion (>60%) among inpatients aged 0-14 years and 65 years and older than the rest, while the proportion of fractures caused by road traffic injuries was higher in inpatients aged 15-69 years (>25%). In addition, the proportion of fractures caused by both mechanical and cutting/perforating/firearm injuries were higher among inpatients aged 0 to 4 years and 20 to 59 years, and the proportion of blunt/impact injuries higher in those aged 15 to 59 years, as shown in **Figure 3**. There were also gender differences in the distribution of causes of injuries in various age groups. For example, compared with men, women will enter the age when they are prone to fractures due to falls earlier.

**Table 2:** Age and gender-stratified overview of causes of traumatic fractures.

Cause of injury, N (%)	Under 15 years		15-29 years		30-44 years		45-59 years		60-74 years		Over 75 years	
	Male	Female	Male	Female	Male	Female	Male	Female	Male	Female	Male	Female
Traffic accident	12873(14.48)	6812(16.21)	28456(27.08)	13293(39.71)	51222(23.17)	29527(37.60)	86101(24.94)	72034(32.80)	59921(30.64)	52600(20.38)	13822(14.74)	13269(6.83)
Fall	66517(74.84)	31078(73.96)	49559(47.17)	15766(47.10)	110619(50.05)	35719(45.49)	175564(50.86)	119660(54.49)	106567(54.50)	176293(68.30)	71432(76.19)	160879(82.85)
Cutting/Perforating/ Firearm injury	311(0.35)	128(0.30)	882(0.84)	122(0.36)	1311(0.59)	349(0.44)	1360(0.39)	477(0.22)	441(0.23)	213(0.08)	51(0.05)	52(0.03)
Blunt/Impact injury	2190(2.46)	811(1.93)	8280(7.88)	930(2.78)	19751(8.94)	3361(4.28)	27595(7.99)	6472(2.95)	7428(3.80)	3312(1.28)	793(0.85)	969(0.50)
Animal attack	30(0.03)	16(0.04)	70(0.07)	11(0.03)	152(0.07)	44(0.06)	366(0.11)	181(0.08)	232(0.12)	201(0.08)	39(0.04)	68(0.04)
Explosion/Burn injury	35(0.04)	14(0.03)	193(0.18)	23(0.07)	446(0.20)	71(0.09)	647(0.19)	135(0.06)	178(0.09)	37(0.01)	14(0.01)	17(0.01)
Mechanical injury	2486(2.80)	1292(3.07)	9153(8.71)	1090(3.26)	22667(10.26)	4453(5.67)	32106(9.30)	7221(3.29)	8693(4.45)	2182(0.85)	658(0.70)	615(0.32)
Other reasons	4437(4.99)	1867(4.44)	8482(8.07)	2239(6.69)	14860(6.72)	5000(6.37)	21439(6.21)	13419(6.11)	12093(6.18)	23273(9.02)	6948(7.41)	18305(9.43)
Total	88879(100%)	42018(100%)	105075(100%)	33474(100%)	221028(100%)	78524(100%)	345178(100%)	219599(100%)	195553(100%)	258111(100%)	93757(100%)	194174(100%)

### 3.4 Common diagnoses and procedures

Femoral neck fractures and intertrochanteric fractures rounded out the top two, with the number of 138377 and 110898 respectively, totaling approximately 12% of all cases. Other vulnerable body parts were the shoulder, knee, ankle, wrist, spine, and ribs. Of all reported cases, a sum of 1506508 inpatients underwent surgery, accounting for 74.39% of total. The top 10 most common diagnoses of traumatic fractures and the top 20 surgical procedures are shown in the online **Supplementary Tables 4** and **5**.

### 3.5 In-Hospital Mortality

In this study we collected 2448 cases of death. The in-hospital mortality of inpatients with traumatic fractures was 1.209‰ and showed an escalating trend with age, as shown in **Table 1**. The in-hospital mortality was higher in males than in females (1.299‰ vs. 1.100‰). There were differences in the distribution of mortality rates by gender in each age group. Female inpatients had higher in-hospital mortality than males between 0 and 34 years of age, and the opposite was true above 35 years of age. The in-hospital mortality was highest in Northeast China (2.338‰) and lowest in East China (0.999‰), as shown in **Table 1** and **Figure 2**. Road traffic injuries resulted in higher in-hospital mortality than any other causes (1.768‰). Among inpatients with traumatic fractures due to road traffic injuries, falls, explosion/burn injuries, and mechanical injuries, there were far more male than female patients who died during hospitalization.

### 3.6 Hospitalization Costs

In 2020, the total hospitalization cost of traumatic fractures in tertiary public hospitals nationwide exceeded ¥40 billion (about $5.5 billion). The median hospitalization cost for all inpatients was ¥20,900 or $3056 (IQR ¥7,700-¥37,700 or $1126-$5512), with ¥19,200 ($2,807) and ¥22,900 ($3348) for males and females, respectively. There was a gradual upward trend in hospitalization costs with increasing age. Inpatients caused by falls had the highest hospitalization cost (median ¥22,200 or $3,245), as shown in **Table 1** and **Figure 2**.

### 3.7 Length of stay(LOS)

The median LOS for both male and female inpatients with traumatic fractures was 10 (IQR 6-16) days. Inpatients aged 45-54 year-old had the longest LOS (median 11 days) and those aged 0-4 year-old had the shortest LOS (median 5 days). In addition, inpatients with traumatic fractures caused by road traffic injuries had the longest LOS (median 13 days), as shown in **Table 1** and **Figure 2**.

## 4 Discussion

This is the first retrospective study to investigate the admission rate, in-hospital mortality and economic burden of traumatic fractures in China. Our study found that: (a) more than 2 million patients suffer from traumatic fractures and need hospitalization annually in China, with hip fracture being the most common diagnosis. (b) The admission rate, in-hospital mortality, hospitalization costs and length of stay of inpatients with traumatic fractures in the Chinese population all increases with age, which means fractures in the elderly should be paid more attention. (c) Falls and road traffic injuries are the main causes of traumatic fractures, and road traffic injuries caused a highest in-hospital mortality.

Globally, there are large regional differences in the incidence of traumatic fractures, which are related to socio-cultural factors such as economic level and lifestyles (23). In 1990s, Norway reported an annual incidence rate of 2.28% for fractures in the whole population (11), and Australia had an annual incidence rate of 1.99% for adults over 35 years old (12). In 2008, Donaldson et al. (9) calculated an overall annual fracture incidence of 3.6% in England. All these data are much higher than the result of this study, suggesting that the incidence of fractures may be higher in developed countries than in China. The data from GBD 2019 demonstrated that in 2019 Australia, Central Europe and Eastern Europe have the highest age-standardized incidence rate of fractures, and East Asia, including China, has a lower incidence of fractures, while sub-Saharan Africa has the lowest (2). By reporting age- and gender-specific incidence rates, a few of studies described a rising trend of fracture incidence with age (4, 5, 10, 12). Among young patients, male usually outnumbered female, while among the middle-aged and the elderly, the male-to-female ratio reversed, which are consistence with our finding. Curtis et al. (10) documented that the fracture incidence in high-income countries followed a bimodal age-sex distribution, peaking in young male due to high-energy trauma and in old female with osteoporotic fragility fractures. However, our research found that the incidence of traumatic fracture among the young adults in China was much lower, which can probably be attributed to China’s recent efforts to promote the trauma prevention and treatment system.

Earlier epidemiological studies on fractures in China usually focused on a certain type of population, region or disease (17, 24–26). The China National Fracture Study in 2015 reported that the annual incidence of traumatic fractures in China was 3.21 per 1000 persons based on a survey for over 500,000 community residents (14). However, the study did not investigate the dead cases and the economic burden. Since 2020, the global pandemic of COVID-19 has severely impacted health care services. Lv et al. (16) found that during the COVID-19 epidemic, traumatic fractures caused by low-energy trauma in the elderly population increased significantly compared to previous years, while fractures caused by road traffic injuries decreased, which may be related to epidemic prevention measures such as strict home confinement and reduction of unnecessary out-of-home travel. Therefore, in the context of the COVID-19 pandemic, a new survey on traumatic fractures in China has a very positive effect on the formulation of public health policies.

This study covered the majority of tertiary public hospitals in China, ensuring the representativeness of the sample. It is estimated in this study that 1.437 per 1000 people in China suffered traumatic fractures requiring hospitalization in 2020. The incidence of traumatic fractures is higher in men than in women, and higher in the elderly than in the young. We noticed that 60 years of age is a critical point for the inpatients of traumatic fractures. Before this age, there are more cases and a higher incidence for male inpatients than for females. After the age of 60, the opposite is true. Between the age of 50 to 69 years, the age-incidence curve for male inpatients hit a plateau, and it rose again after 70 years old, whereas the age-incidence curve for female inpatients maintained a rising trend throughout this period. We speculate that this is because men are usually energetic, aggressive, and occupied in more heavy and hazardous works, as well as preferring to participate in vigorous sports, resulting in a higher incidence of traumatic fractures at a young age. After the age of 50, the body physiological senescence makes men have less opportunity to participate in heavy or dangerous work, thus the incidence of traumatic fractures stops rising. For the middle-aged and elderly, osteoporosis is an important risk factor for traumatic fractures. The China Osteoporosis Prevalence Study showed that in the Chinese population, the prevalence of osteoporosis increased significantly in men and women after the age of 70 and 50 years, respectively, and the prevalence in women was significantly higher than that in men since the age of 50 years (27). This showed a consistent trend with the relationship between the incidence of traumatic fractures and age. Therefore, we infer that traumatic fractures in male patients occurring after the age of 70 years tend to be fragility fractures due to low-energy trauma, and for women, the age prone to developing osteoporotic fractures may be as early as 50 years.

We found that falls and road traffic injuries were the top two causes of injury in all age groups, which is consistent with the results of previous studies (14–16). We also summarized the tendency of injury causes in different age groups, for example, the age groups prone to traumatic fractures due to falls were 0-14 years and 65 years or older, the age groups prone to traumatic fractures due to road traffic injuries were 15-69 years, the age groups prone to traumatic fractures due to mechanical injuries or cutting/penetrating/firearm injuries were 0-4 years and 20-59 years, and the age groups prone to traumatic fractures due to blunt/impact injuries were 15-59 years. In conclusion, the distribution of causes of injuries tends to vary in each age group. Newborns and infants (less than 5 years old) do not have complete cognitive and behavioral abilities, and are vulnerable to accidental injuries such as falls, penetrating injuries, and mechanical injuries, so their guardians should be more vigilant to strengthen prevention. The young and middle-aged (15-59 years old) need involve in social life and engage in various jobs, so the factors that cause traumatic fractures in this age group become diversified and may come more from travel scenarios and work scenarios. The elderly (60 years old and above) gradually return to a simpler lifestyle and have a limited activity of daily living, which, combined with the effects of osteoporosis, make them more susceptible to fragility fractures caused by low-energy trauma such as falls.

In our study, the aged inpatients had a higher in-hospital mortality, and consumed more hospitalization costs. Obviously, aging of population exacerbates the disease burden of traumatic fractures. According to the data disclosed by China’s National Health Commission, the country is expected to enter a stage of heavy population aging, referring to more than 30% of the population over 60 years old, by around 2035 (28). We can extrapolate that a larger number of incidences of osteoporotic fragility fractures may occur in the next 15 years. Therefore, taking measures to prevent osteoporotic fractures and improve the ability of early diagnosis and treatment of osteoporosis will be one of the key tasks in the field of health care in the future.

Road traffic injuries led to a highest in-hospital mortality in our research, because the high-energy traumas that can easily bring about multiple injuries involving head, chest, abdomen, or pelvis, resulting in serious even life-threatening complications. Official statistics show that road traffic injuries are the leading cause of injury deaths among Chinese residents, with about 60,000 people dying each year as a result. Recent years, trauma medicine in China has been greatly developed under the support by authority. The model of a “closed-loop regional trauma treatment system with the general hospital as the core” has gradually matured, which emphasizes on standardizing the treatment process of severe trauma and strengthening the information communication among the pre-hospital emergency team, emergency medical team and specialized treatment team. It has successfully changed the status quo and process of severe trauma treatment in China, shortening the average treatment time by 50% and reducing the mortality rate by 39.6% year-on-year (29–32). On the other hand, The China government has made efforts to improve traffic safety through a series of administrative or legal measures, including investing heavily in improving public transportation infrastructure, building a high-speed railroad network, enacting the Road Traffic Safety Law, and incorporating drunk driving into criminal law (31, 33, 34). However, we should admit that the heavy burden resulted from traumatic fractures caused by road traffic injuries still needs more attention. Enhancing the safety awareness of traffic participants and developing safer high-tech traffic technology should be the direction of the whole society in the future.

The strength of this study Is the pooling of data from a large sample of 31 provinces, municipalities, and autonomous regions in mainland China. This study first compared admission rate, in-hospital mortality, hospitalization costs, and length of stay for traumatic fractures among different ages, regions, and causes of injury, providing a preliminary picture of the overall burden of disease of traumatic fractures in China. Admittedly, there are some shortcomings in this study. First, the data of inpatients only do not yet reflect the full picture of the epidemiological characteristics of patients with traumatic fractures in China, because many traumatic fractures were treated in outpatient clinics or emergency departments, and the corresponding medical records are lacking in the HQMS system. Therefore, the number of cases of traumatic fractures in China may be underestimated. Second, the data obtained from the HQMS system in this study were coarse in granularity and could not be used to conduct further statistical analysis, which is a result of the jurisdiction of the Data Security Law. Third, this study covers only a single year of data and fails to describe the trends in the data. This issue will be gradually resolved as our study progresses.

In conclusion, traumatic fractures pose a serious public health burden in China today, and these fractures are mostly caused by falls and traffic accidents. It is important to promote the number, coverage, and treatment level of trauma centers all over the nation. But the key to reducing the incidence of traumatic fractures in the future, reducing the burden of disease and improving the quality of life of patients still lies in the prevention of trauma, and the country should invest more to further improve prevention strategies and enhance the health of the whole population.

## Data availability statement

The original contributions presented in the study are included in the article/**Supplementary Material**. Further inquiries can be directed to the corresponding author.

## Ethics statement

The study was approved by Institutional Review Board of China National Center for Trauma Medicine, Peking University People’s Hospital (No. 2022PHB306).

## Author contributions

BH, YW collected the data, analyzed the data and wrote the manuscript. HW, CW, FJ, JL performed statistical analysis and helped edit the manuscript. BH prepared the figures. LG, YS contributed to the data collection and analysis. BJ and DZ obtained funding, designed the study, analyzed the data, and made critical revisions of the manuscript. All authors contributed to the article and approved the submitted version.
