# Supplementary material for: Epidemiology and the economic burden of traumatic fractures in China: A population-based study
Source: Front Endocrinol (Lausanne). 2023 Jan 24;14:1104202. doi: 10.3389/fendo.2023.1104202 (PMC9902367; doi:10.3389/fendo.2023.1104202)
Supplement: Supplementary file 1 [file Table_1.docx]

**Supplementary table 1** ICD-10 diagnosis codes for traumatic fractures

| **ICD-10 code** | **Diagnose** |
| --- | --- |
| S11.800x011 | Open neck injury with cervical fracture |
| S12.000 | Fracture of first cervical vertebra |
| S12.000x002 | Fracture of atlas |
| S12.010 | Open fracture of first cervical vertebra |
| S12.100 | Fracture of second cervical vertebra |
| S12.100x001 | Fracture of anterior and posterior arch of atlas (Jefferson fracture) |
| S12.100x002 | Fracture of axis |
| S12.100x003 | Fracture of axis pedicle fracture(Hangman fracture) |
| S12.110 | Open fracture of second cervical vertebra |
| S12.200 | Fracture of other specified cervical vertebra |
| S12.200x001 | Fracture of odontoid process of axis |
| S12.200x002 | Axis fracture with dislocation (Hangman fracture) |
| S12.200x011 | C3 cervical vertebra fracture |
| S12.200x021 | C4 cervical vertebra fracture |
| S12.200x031 | C5 cervical vertebra fracture |
| S12.200x041 | C6 cervical vertebra fracture |
| S12.200x051 | C7 cervical vertebra fracture |
| S12.210 | Open fracture of specified cervical vertebra |
| S12.700 | Multiple fractures of cervical vertebras |
| S12.700x001 | Multiple fractures of cervical vertebras |
| S12.710 | Multiple open fractures of cervical vertebras |
| S12.800 | Fracture of neck, other part |
| S12.810 | Open fracture of neck, other part |
| S12.900 | Fracture of neck |
| S12.900x001 | Fracture of cervical vertebra |
| S12.900x003 | Fracture of cervical nerve arch |
| S12.900x004 | Fracture of spinous process of cervical vertebra |
| S12.900x005 | Fracture of cervical transverse process |
| S12.900x006 | Fracture of cervical vertebra arch |
| S12.910 | Open fracture of cervical vertebra |
| S21.800x011 | Open injury of chest with fracture |
| S22.000 | Fracture of thoracic vertebra |
| S22.000x003 | Compression fracture of thoracic vertebra |
| S22.000x005 | Fracture of thoracic spinal nerve arch |
| S22.000x006 | Fracture of spinous process of thoracic vertebra |
| S22.000x007 | Fracture of transverse process of thoracic vertebra |
| S22.000x009 | Fracture of thoracic vertebral arch |
| S22.000x011 | T1/T2 thoracic vertebra fracture |
| S22.000x021 | T3/T4 thoracic vertebra fracture |
| S22.000x031 | T5/T6 thoracic vertebra fracture |
| S22.000x041 | T7/T8 thoracic vertebra fracture |
| S22.000x051 | T9/T10 thoracic vertebra fracture |
| S22.000x061 | T11/T12 thoracic vertebra fracture |
| S22.010 | Open fracture of thoracic vertebra |
| S22.100 | Multiple fractures of thoracic vertebras |
| S22.110 | Multiple open fractures of thoracic vertebras |
| S22.200 | Fracture of sternum |
| S22.210 | Open fracture of sternum |
| S22.300 | Fracture of rib |
| S22.300x011 | Fracture of first rib |
| S22.310 | Open fracture of rib |
| S22.400 | Multiple fractures of ribs |
| S22.400x011 | Multiple fractures of ribs with first rib fracture |
| S22.400x021 | Two rib fractures without the first rib fracture |
| S22.400x031 | Three rib fractures without first rib fracture |
| S22.400x041 | More than four rib fractures without the first rib fracture |
| S22.410 | Multiple open fractures of ribs |
| S22.800 | Fracture of other parts of bony thorax |
| S22.810 | Open fracture of specific part of bony thorax |
| S22.900 | Fracture of bony thorax |
| S22.900x001 | Fracture of thorax |
| S22.910 | Open fracture of bony thorax |
| S31.800x011 | Open injury of low back with fracture |
| S31.800x012 | Open injury of pelvis with fracture |
| S32.000 | Fracture of lumbar vertebra |
| S32.000x002 | Compression fracture of thoracic vertebra |
| S32.000x011 | L1 lumbar vertebra fracture |
| S32.000x021 | L2 lumbar vertebra fracture |
| S32.000x031 | L3 lumbar vertebra fracture |
| S32.000x041 | L4 lumbar vertebra fracture |
| S32.000x051 | L5 lumbar vertebra fracture |
| S32.010 | Open fracture of lumbar vertebra |
| S32.100 | Fracture of sacrum |
| S32.110 | Open fracture of sacrum |
| S32.200 | Fracture of coccyx |
| S32.210 | Open fracture of coccyx |
| S32.300 | Fracture of ilium |
| S32.310 | Open fracture of ilium |
| S32.400 | Fracture of acetabulum |
| S32.410 | Open fracture of acetabulum |
| S32.500 | Fracture of pubis |
| S32.500x002 | Fracture of pubic branch |
| S32.500x003 | Fracture of pubic symphysis |
| S32.510 | Open fracture of pubis |
| S32.700 | Multiple fractures of lumbar spine and pelvis |
| S32.701 | Multiple fractures of pelvis |
| S32.702 | Multiple fractures of lumbar spine |
| S32.710 | Multiple open fractures of lumbar spine and pelvis |
| S32.711 | Multiple open fractures of pelvis |
| S32.712 | Multiple open fractures of lumbar spine |
| S32.800 | Fracture of other and unspecified parts of lumbar spine and pelvis |
| S32.800x021 | Fracture of lumbosacral spinous process |
| S32.800x022 | Fracture of lumbosacral transverse process |
| S32.800x023 | Fracture of lumbosacral arch |
| S32.800x024 | Fracture of Lumbosacral vertebra |
| S32.800x091 | Fracture of symphysis pelvis |
| S32.800x092 | Pelvic lateral compression fracture |
| S32.800x093 | Pelvic open-book fracture |
| S32.800x094 | Pelvic vertical shear fracture |
| S32.800x095 | Malgaigne fracture |
| S32.801 | Fracture of ischium |
| S32.802 | Fracture of pelvic |
| S32.803 | Fracture of lumbosacral spinal |
| S32.810 | Open fracture of specific part of lumbar spine and pelvis |
| S32.811 | Open fracture of ischium |
| S32.812 | Open fracture of pelvic |
| S32.813 | Open fracture of lumbosacral spinal |
| S41.800x011 | Open injury of shoulder with fracture |
| S41.800x012 | Open injury of upper arm with fracture |
| S42.000 | Fracture of clavicle |
| S42.000x011 | Fracture of sternal end of clavicle |
| S42.000x021 | Fracture of clavicular shaft |
| S42.000x031 | Fracture of acromion end of clavicle |
| S42.000x091 | Multiple fractures of clavicle |
| S42.010 | Open fracture of clavicle |
| S42.100 | Fracture of scapula |
| S42.100x011 | Fracture of scapular body |
| S42.100x021 | Fracture of acromion |
| S42.100x031 | Fracture of coracoid process of scapula |
| S42.100x041 | Fracture of scapular neck and glenoid |
| S42.100x042 | Fracture of glenoid |
| S42.100x091 | Multiple fractures of scapula |
| S42.110 | Open fracture of scapula |
| S42.200 | Fracture of upper end of humerus |
| S42.200x001 | Fracture of proximal humerus |
| S42.200x031 | Fracture of anatomical neck of humerus |
| S42.200x041 | Fracture of greater tubercle of humerus |
| S42.200x091 | Fracture of lesser tubercle of humerus |
| S42.200x092 | Multiple fractures of proximal humerus |
| S42.200x101 | Fracture of humeral neck |
| S42.202 | Fracture of surgical neck of humerus |
| S42.203 | Fracture of humeral head |
| S42.210 | Open fracture of upper humerus |
| S42.300 | Fracture of humeral shaft |
| S42.300x002 | Multiple fractures of humeral shaft |
| S42.301 | Fracture of humerus |
| S42.310 | Open fracture of humeral shaft |
| S42.311 | Open fracture of humerus |
| S42.400 | Fracture of lower end of humerus |
| S42.400x001 | Fracture of distal humerus |
| S42.400x041 | Fracture of medial epicondyle of humerus |
| S42.400x042 | Fracture of lateral epicondyle of humerus |
| S42.400x051 | T-shaped fracture of distal humerus |
| S42.400x091 | Multiple fractures of distal humerus |
| S42.400x092 | Fracture of trochlear of humerus |
| S42.400x093 | Fracture of capitulum of humerus |
| S42.401 | Supracondylar fracture of humerus |
| S42.402 | Fracture of lateral condyle of humerus |
| S42.403 | Intercondylar fracture of humerus |
| S42.404 | Fracture of medial condyle of humerus |
| S42.410 | Open fracture of lower humerus |
| S42.700 | Multiple fractures of clavicle, scapula and humerus |
| S42.710 | Multiple open fractures of clavicle, scapula and humerus |
| S42.800 | Fracture of other parts of shoulder and upper arm |
| S42.810 | Open fracture of specific part of shoulder and upper arm |
| S42.900 | Fracture of shoulder girdle |
| S42.910 | Open fracture of shoulder |
| S51.800x011 | Open injury of forearm with fracture |
| S52.000 | Fracture of upper end of ulna |
| S52.000x001 | Fracture of elbow joint |
| S52.000x002 | Fracture of proximal ulna |
| S52.000x011 | Fracture of olecranon of ulna |
| S52.000x021 | Fracture of coronoid process of ulna |
| S52.000x091 | Multiple fractures of proximal ulna |
| S52.001 | Olecranon fracture |
| S52.002 | Monteggia fracture dislocation |
| S52.010 | Open fracture of upper end of ulna |
| S52.011 | Open fracture of olecranon |
| S52.100 | Fracture of upper radius |
| S52.100x001 | Fracture of proximal radius |
| S52.100x091 | Multiple fractures of proximal radius |
| S52.101 | Fracture of radial head |
| S52.102 | Fracture of radial neck |
| S52.110 | Open fracture of upper radius |
| S52.200 | Fracture of ulnar shaft |
| S52.200x011 | Monteggia fracture |
| S52.201 | Ulnar fracture |
| S52.210 | Open fracture of ulnar shaft |
| S52.211 | Open fracture of ulna |
| S52.300 | Fracture of radial shaft |
| S52.300x011 | Galeazzi fracture |
| S52.310 | Open fracture of radial shaft |
| S52.400 | Fracture of ulna and radius diaphysis |
| S52.400x001 | Fracture of ulna and radius shaft |
| S52.410 | Open fracture of ulna and radius shaft |
| S52.500 | Fracture of lower end of radius |
| S52.500x001 | Fracture of distal radius |
| S52.500x002 | Fracture of styloid process of radius |
| S52.500x011 | Colles Fracture |
| S52.500x021 | Barton fracture |
| S52.500x022 | Smith fracture |
| S52.500x091 | Intraarticular fracture of radius |
| S52.501 | Flexion fracture of distal radius |
| S52.502 | Straight fracture of lower radius |
| S52.510 | Open fracture of distal radius |
| S52.600 | Fracture of distal radius and ulna |
| S52.600x001 | Distal ulna fracture with distal radius fracture |
| S52.600x002 | Ulnar styloid process fracture with distal radius fracture |
| S52.610 | Open fracture of distal radius and ulna |
| S52.700 | Multiple fractures of forearm |
| S52.701 | Closed fracture of radius and ulna |
| S52.710 | Open multiple fractures of forearm |
| S52.711 | Open fracture of radius and ulna |
| S52.800 | Fracture of other parts of forearm |
| S52.801 | Fracture of radius |
| S52.802 | Fracture of styloid process of ulna |
| S52.803 | Fracture of ulnar head |
| S52.804 | Fracture of lower end of ulna |
| S52.810 | Open fracture of forearm |
| S52.811 | Open fracture of radius |
| S52.812 | Open fracture of styloid process of ulna |
| S52.813 | Open fracture of ulnar head |
| S52.814 | Open fracture of lower end of ulna |
| S52.900 | Fracture of forearm |
| S61.800x011 | Open injury of wrist and hand with fracture |
| S61.800x012 | Open injury of hand with fracture |
| S61.800x013 | Open injury of wrist with fracture |
| S62.000 | Fracture of scaphoid |
| S62.000x001 | Fracture of scaphoid of wrist |
| S62.010 | Open fracture of scaphoid of hand |
| S62.100 | Fracture of carpus, other special |
| S62.100x011 | Fracture of lunate |
| S62.100x021 | Fracture of triangular |
| S62.100x031 | Fracture of pisiform |
| S62.100x041 | Fracture of trapezium |
| S62.100x051 | Fracture of trapezoid |
| S62.100x061 | Fracture of capitate bone |
| S62.100x071 | Fracture of hamate |
| S62.100x091 | Multiple fracture of carpus |
| S62.101 | Fracture of carpal bone |
| S62.110 | Open fracture of specific carpal bone |
| S62.111 | Open fracture of carpus |
| S62.200 | Fracture of the first metacarpal bone |
| S62.200x011 | Fracture of the first metacarpal base |
| S62.200x021 | Fracture of the first metacarpal shaft |
| S62.200x031 | Fracture of the first metacarpal neck |
| S62.200x041 | Fracture of the first metacarpal head |
| S62.201 | Bennett fracture |
| S62.210 | Open fracture of the first metacarpal bone |
| S62.300 | Fracture of metacarpal, others |
| S62.300x011 | Fracture of metacarpal base |
| S62.300x021 | Fracture of metacarpal shaft |
| S62.300x031 | Fracture of metacarpal neck |
| S62.300x041 | Fracture of metacarpal head |
| S62.301 | Fracture of metacarpal |
| S62.310 | Open fracture of specific metacarpal |
| S62.311 | Open fracture of metacarpal |
| S62.400 | Multiple fractures of metacarpal |
| S62.410 | Open multiple fractures of metacarpal |
| S62.500 | Fracture of thumb |
| S62.500x011 | Fracture of proximal segment of thumb |
| S62.500x021 | Fracture of distal segment of thumb |
| S62.510 | Open fracture of thumb |
| S62.600 | Finger fracture, others |
| S62.600x011 | Fracture of proximal phalanx |
| S62.600x021 | Fracture of middle phalanx |
| S62.600x031 | Fracture of distal phalanx |
| S62.610 | Open fracture of specific phalanx |
| S62.611 | Open fracture of phalanx |
| S62.700 | Multiple fractures of phalanx |
| S62.710 | Open multiple fractures of phalanx |
| S62.800 | Fractures of wrist and hand, part unspecified |
| S62.801 | Hand fracture |
| S62.802 | Fracture of phalanx |
| S62.810 | Open fracture of wrist and hand, part unspecified |
| S62.811 | Open fracture of hand |
| S71.800x011 | Open injury of hip with fracture |
| S71.800x012 | Open injury of thigh with fracture |
| S72.000 | Femoral neck fracture |
| S72.000x011 | Intracystic fracture of femoral joint |
| S72.000x031 | Subcapitular fracture of femoral neck |
| S72.000x041 | Transcervical fracture of femoral neck |
| S72.000x051 | Femoral neck basal fracture |
| S72.000x081 | Femoral head fracture |
| S72.000x082 | Hip fracture |
| S72.010 | Open femoral neck fracture |
| S72.100 | Transtrochanteric fracture |
| S72.100x001 | Fracture of greater trochanter of femur |
| S72.100x002 | Fracture of lesser trochanter of femur |
| S72.100x011 | Intertrochanteric fracture of femur |
| S72.101 | Intertrochanteric fracture of femur |
| S72.110 | Open intertrochanteric fracture of femur |
| S72.200 | Subtrochanteric fracture |
| S72.200x001 | Subtrochanteric fracture of femur |
| S72.210 | Open subtrochanteric fracture of femur |
| S72.300 | Femoral shaft fracture |
| S72.310 | Open fracture of femoral shaft |
| S72.400 | Fracture of lower femur |
| S72.400x001 | Fracture of distal femur |
| S72.400x012 | Fracture of medial femoral condyle |
| S72.400x013 | Fracture of lateral femoral condyle |
| S72.400x031 | Supracondylar fracture of femur |
| S72.400x041 | Intercondylar fracture of femur |
| S72.401 | Fracture of femoral condyle |
| S72.410 | Open fracture of lower femur |
| S72.700 | Multiple fractures of the femur |
| S72.710 | Open multiple fractures of the femur |
| S72.800 | Fractures of femur, other parts |
| S72.810 | Open fracture of specific part of femur |
| S72.900 | Fractures of femur |
| S72.910 | Open fractures of femur |
| S81.800x011 | Open injury of crus with fracture |
| S82.000 | Fracture of patella |
| S82.000x003 | Cuff avulsion fracture of patella |
| S82.000x004 | Sleeve fracture of patella |
| S82.010 | Open fracture of patella |
| S82.100 | Fracture of upper tibia |
| S82.100x011 | Fracture of proximal tibia and fibula |
| S82.100x012 | Tibial plateau fracture with fibula fracture |
| S82.100x081 | Fracture of proximal tibia |
| S82.100x084 | Fracture of tibial condyle |
| S82.100x085 | Fracture of tibial intercondylar eminence |
| S82.100x086 | Fracture of lateral tibial condyle |
| S82.100x087 | Tibial plateau fracture |
| S82.100x088 | Tibial plateau fracture with intercondylar fracture |
| S82.100x089 | Fracture of tibial tuberosity |
| S82.101 | Closed tibial plateau fracture |
| S82.102 | Fracture of tibial head |
| S82.110 | Open fracture of the upper tibia |
| S82.111 | Open fracture of tibial head |
| S82.200 | Fracture of tibial shaft |
| S82.200x011 | Fracture of tibia shaft with fracture of fibula |
| S82.200x081 | Fracture of tibial shaft |
| S82.201 | Fracture of tibiofibular shaft |
| S82.202 | Fracture of fibula |
| S82.203 | Closed fracture of tibia and fibula |
| S82.210 | Open fracture of tibial shaft |
| S82.211 | Open fracture of tibial |
| S82.212 | Open fracture of tibiofibular shaft |
| S82.300 | Fracture of lower tibia |
| S82.300x011 | Fracture of distal tibia with fracture of fibula |
| S82.300x081 | Fracture of distal tibia |
| S82.300x083 | Pilon fracture |
| S82.301 | Fracture of lower tibia and fibula |
| S82.310 | Open fracture of lower tibia |
| S82.311 | Open fracture of lower tibia and fibula |
| S82.400 | Fracture of fibula, only |
| S82.400x001 | Fracture of fibula |
| S82.400x011 | Fracture of proximal fibula |
| S82.400x012 | Fracture of fibular head |
| S82.400x013 | Fracture of fibular neck |
| S82.400x014 | Fracture of fibular capitulum |
| S82.400x091 | Multiple fracture of fibula |
| S82.401 | Fracture of fibular shaft |
| S82.410 | Open fracture of fibula |
| S82.411 | Open fracture of fibular shaft |
| S82.500 | Fracture of medial malleolus |
| S82.501 | Fracture of tibia involving ankle joint |
| S82.510 | Open fracture of medial malleolus |
| S82.600 | Fracture of lateral malleolus |
| S82.601 | Fracture of fibula involving ankle joint |
| S82.610 | Open fracture of lateral malleolus |
| S82.700 | Multiple fractures of lower leg |
| S82.710 | Open multiple fractures of lower leg |
| S82.800 | Fracture of other parts of the lower leg |
| S82.800x081 | Fracture of ankle |
| S82.800x082 | Fracture of ankle joint |
| S82.801 | Trimalleolar fracture of ankle |
| S82.802 | Bimalleolar fracture of ankle |
| S82.803 | Closed fracture of ankle |
| S82.810 | Open fracture of specific part of lower leg |
| S82.811 | Open trimalleolar fracture of ankle |
| S82.812 | Open bimalleolar fracture of ankle |
| S82.900 | Fracture of lower leg |
| S82.910 | Open fracture of lower leg |
| S91.300x811 | Open injury of ankle and foot with fracture |
| S91.300x812 | Open injury of ankle with fracture |
| S91.300x813 | Open injury of foot with fracture |
| S92.000 | Fracture of calcaneus |
| S92.010 | Open fracture of calcaneus |
| S92.100 | Fracture of talus |
| S92.100x003 | Fracture of talus body |
| S92.101 | Fracture of talus neck |
| S92.110 | Open fracture of talus |
| S92.200 | Fracture of tarsal bone, others |
| S92.200x001 | Fracture of tarsal bone |
| S92.200x011 | Fracture of scaphoid of foot |
| S92.200x081 | Fracture of tarsal joint |
| S92.201 | Fracture of cuboid bone |
| S92.202 | Fracture of scaphoid bone of foot |
| S92.203 | Fracture of cuneiform bone |
| S92.210 | Open fracture of specific tarsal bone |
| S92.300 | Fracture of metatarsal |
| S92.300x001 | Fracture of tarsometatarsal joint |
| S92.300x003 | Fracture of metatarsal base |
| S92.310 | Open fracture of metatarsal |
| S92.400 | Fracture of great toe |
| S92.410 | Open fracture of great toe |
| S92.500 | Fracture of phalanx, others |
| S92.500x001 | Fracture of phalanx |
| S92.510 | Open fracture of specific phalanx |
| S92.700 | Multiple fractures of foot |
| S92.700x001 | Multiple fractures of foot bone |
| S92.710 | Open multiple fractures of foot |
| S92.900 | fracture of foot |
| S92.910 | Open fracture of foot |
| S93.300x031 | Fracture-dislocation of tarsometatarsal joint(Lisfranc injury) |
| T02.000 | Fractures involving head and neck |
| T02.000x001 | Fractures of head and neck |
| T02.010 | Open fractures of head and neck |
| T02.100 | Fracture involving chest with lower back and pelvis |
| T02.100x001 | Multiple fractures of trunk |
| T02.110 | Open multiple fractures of trunk |
| T02.200 | Fracture involving multiple parts of single upper limb |
| T02.200x001 | Multiple fractures of single upper limb |
| T02.210 | Open multiple fractures of single upper limb |
| T02.300 | Fracture involving multiple parts of single lower limb |
| T02.300x001 | Multiple fractures of single lower limb |
| T02.310 | Open multiple fractures of single lower limb |
| T02.400 | Fracture involving multiple parts of both upper limbs |
| T02.400x001 | Multiple fractures of both upper limbs |
| T02.410 | Open multiple fractures of both upper limbs |
| T02.500 | Fracture involving multiple parts of both lower limbs |
| T02.500x001 | Multiple fractures of both lower limbs |
| T02.510 | Open multiple fractures of both lower limbs |
| T02.600 | Fracture involving upper limb with multiple parts of lower limb |
| T02.600x001 | Upper limb with multiple fractures of lower limb |
| T02.600x011 | Multiple open fractures of upper limb with lower limb |
| T02.610 | Open multiple fractures of limbs |
| T02.700 | Fractures involving chest with lower back, pelvis and limbs |
| T02.700x001 | Fractures of thorax, lower back, pelvis and limbs |
| T02.700x021 | Fractures of pelvis and lower limbs |
| T02.710 | Open fractures of thorax, lower back, pelvis and limbs |
| T02.800 | Fractures involving other complex parts of the body |
| T02.800x001 | Fractures of compound part of the body |
| T02.810 | Open fractures of the specific compound part of body |
| T02.900 | Multiple fractures |
| T02.910 | Open multiple fractures |
| T08.x00 | Fracture of spine |
| T08.x10 | Open fracture of spine |
| T10.x00 | Fracture of upper limb |
| T10.x10 | Open fracture of upper limb |
| T12.x00 | Fracture of lower limb |
| T12.x10 | Open fracture of lower limb |
| T14.200 | Body fracture |
| T14.210 | Open fracture |

Notes: The following table lists 436 diagnoses and corresponding ICD-10 codes, which we defined as "traumatic fractures". The inclusion and exclusion procedure were as follows: 581 diagnoses containing the word "fracture" were retrieved from the S- and T-segment of ICD-10 (China National Clinical Version 2.0), which were titled “injury, poisoning, and certain other consequences of external causes”, also meant trauma, and "skull fracture "(n=71), "old fracture" (n=58), "sequelae of fracture" (n=7), "cartilage fracture" (n=6), and "internal fixation of fracture" (n=3) were excluded.
