# Supplementary material for: Epidemiology and the economic burden of traumatic fractures in China: A population-based study
Source: Front Endocrinol (Lausanne). 2023 Jan 24;14:1104202. doi: 10.3389/fendo.2023.1104202 (PMC9902367; doi:10.3389/fendo.2023.1104202)
Supplement: Supplementary file 2 [file Table_2.docx]

**Supplementary table 2** The overview of epidemiology characteristics of inpatients with traumatic fractures in China in 2020 stratified by age and gender

| **Age** | **Male** | | | | | | **Female** | | | | | | **Total** | | | | | |
| --- | --- | --- | --- | --- | --- | --- | --- | --- | --- | --- | --- | --- | --- | --- | --- | --- | --- | --- |
|  | Frequency | Proportion,% | Population | Admission rate, per 1000 | Number of Dead case | In-hospital mortality, per 1000 | Frequency | Proportion,% | Population | Admission rate, per 1000 | Number of Dead case | In-hospital mortality, per 1000 | Frequency | Proportion,% | Population | Admission rate, per 1000 | Number of Dead case | In-hospital mortality, per 1000 |
| **0~4** | 19852 | 1.76 | 40969331 | 0.485 | 2 | 0.101 | 13941 | 1.55 | 36914557 | 0.378 | 0 | 0.000 | 33793 | 1.67 | 77883888 | 0.434 | 2 | 0.059 |
| **5~9** | 36633 | 3.25 | 48017458 | 0.763 | 1 | 0.027 | 18896 | 2.11 | 42226598 | 0.447 | 2 | 0.106 | 55529 | 2.74 | 90244056 | 0.615 | 3 | 0.054 |
| **10~14** | 37488 | 3.32 | 45606790 | 0.822 | 11 | 0.293 | 11781 | 1.31 | 39649204 | 0.297 | 4 | 0.340 | 49269 | 2.43 | 85255994 | 0.578 | 15 | 0.304 |
| **15~19** | 29134 | 2.58 | 39053343 | 0.746 | 15 | 0.515 | 8590 | 0.96 | 33630797 | 0.255 | 6 | 0.698 | 37724 | 1.86 | 72684140 | 0.519 | 21 | 0.557 |
| **20~24** | 32781 | 2.9 | 39675995 | 0.826 | 20 | 0.610 | 11275 | 1.26 | 35265680 | 0.320 | 8 | 0.710 | 44056 | 2.18 | 74941675 | 0.588 | 28 | 0.636 |
| **25~29** | 51151 | 4.53 | 48162270 | 1.062 | 31 | 0.606 | 16724 | 1.87 | 43685062 | 0.383 | 12 | 0.718 | 67875 | 3.35 | 91847332 | 0.739 | 43 | 0.634 |
| **30~34** | 81176 | 7.19 | 63871808 | 1.271 | 46 | 0.567 | 26657 | 2.97 | 60273382 | 0.442 | 17 | 0.638 | 107833 | 5.32 | 124145190 | 0.869 | 63 | 0.584 |
| **35~39** | 74654 | 6.61 | 50932037 | 1.466 | 39 | 0.522 | 25984 | 2.9 | 48080895 | 0.540 | 10 | 0.385 | 100638 | 4.97 | 99012932 | 1.016 | 49 | 0.487 |
| **40~44** | 82513 | 7.31 | 47632694 | 1.732 | 50 | 0.606 | 32315 | 3.61 | 45322636 | 0.713 | 12 | 0.371 | 114828 | 5.67 | 92955330 | 1.235 | 62 | 0.540 |
| **45~49** | 118182 | 10.47 | 58191686 | 2.031 | 73 | 0.618 | 55847 | 6.23 | 56033201 | 0.997 | 30 | 0.537 | 174029 | 8.59 | 114224887 | 1.524 | 103 | 0.592 |
| **50~54** | 137610 | 12.19 | 61105470 | 2.252 | 118 | 0.857 | 86288 | 9.63 | 60058826 | 1.437 | 41 | 0.475 | 223898 | 11.06 | 121164296 | 1.848 | 159 | 0.710 |
| **55~59** | 114721 | 10.16 | 50816026 | 2.258 | 109 | 0.950 | 93962 | 10.48 | 50584760 | 1.858 | 50 | 0.532 | 208683 | 10.3 | 101400786 | 2.058 | 159 | 0.762 |
| **60~64** | 82258 | 7.29 | 36871125 | 2.231 | 123 | 1.495 | 88096 | 9.83 | 36511813 | 2.413 | 45 | 0.511 | 170354 | 8.41 | 73382938 | 2.321 | 168 | 0.986 |
| **65~69** | 76329 | 6.76 | 36337923 | 2.101 | 125 | 1.638 | 105155 | 11.73 | 37667637 | 2.792 | 84 | 0.799 | 181484 | 8.96 | 74005560 | 2.452 | 209 | 1.152 |
| **70~74** | 52453 | 4.65 | 24162733 | 2.171 | 120 | 2.288 | 88446 | 9.87 | 25427303 | 3.478 | 76 | 0.859 | 140899 | 6.96 | 49590036 | 2.841 | 196 | 1.391 |
| **75~79** | 38605 | 3.42 | 14752433 | 2.617 | 142 | 3.678 | 75838 | 8.46 | 16486416 | 4.600 | 128 | 1.688 | 114443 | 5.65 | 31238849 | 3.663 | 270 | 2.359 |
| **80~84** | 32625 | 2.89 | 9157003 | 3.563 | 169 | 5.180 | 70218 | 7.83 | 11225875 | 6.255 | 166 | 2.364 | 102843 | 5.08 | 20382878 | 5.046 | 335 | 3.257 |
| **85~** | 30693 | 2.72 | 6100269 | 5.031 | 272 | 8.862 | 66298 | 7.4 | 9317688 | 7.115 | 291 | 4.389 | 96991 | 4.79 | 15417957 | 6.291 | 563 | 5.805 |
| **Total** | 1128858 | 100 | 721416394 | 1.565 | 1466 | 1.299 | 896311 | 100 | 688362330 | 1.302 | 982 | 1.096 | 2025169 | 100 | 1409778724 | 1.437 | 2448 | 1.209 |
