# Supplementary material for: Epidemiology and the economic burden of traumatic fractures in China: A population-based study
Source: Front Endocrinol (Lausanne). 2023 Jan 24;14:1104202. doi: 10.3389/fendo.2023.1104202 (PMC9902367; doi:10.3389/fendo.2023.1104202)
Supplement: Supplementary file 3 [file Table_3.docx]

**Supplementary table 3** The overview of epidemiology characteristics and economic burden of inpatients with traumatic fractures in China in 2020 stratified by province.

| **Region** | **Frequency** | | **Proportion,%** | **Population** | **Admission rate, per 1000** | **Number of Dead case** | **In-hospital mortality, per 1000** | **Length of stay, Median(IQR), Days** | **Hospitalization Cost, Median(IQR), ¥10,000.00** |
| --- | --- | --- | --- | --- | --- | --- | --- | --- | --- |
| **North China** |  | |  |  |  |  |  |  |  |
| Beijing | 39418 | | 1.95 | 21,893,095 | 1.800 | 79 | 2.004 | 7.0 (4.0 to 11.0) | 4.61 (3.04 to 6.27) |
| Tianjin | 26292 | | 1.30 | 13,866,009 | 1.896 | 65 | 2.472 | 9.0 (5.0 to 15.0) | 3.62 (1.27 to 6.29) |
| Hebei | 89353 | | 4.41 | 74,610,235 | 1.198 | 107 | 1.197 | 11.0 (7.0 to 17.0) | 2.45 (1.10 to 4.11) |
| Shanxi | 41313 | | 2.04 | 34,915,616 | 1.183 | 54 | 1.307 | 11.0 (7.0 to 17.0) | 2.45 (1.25 to 3.85) |
| Inner Mongolia | 37585 | | 1.86 | 24,049,155 | 1.563 | 52 | 1.384 | 10.0 (6.0 to 17.0) | 2.21 (0.89 to 3.49) |
| **Northeast China** |  | |  |  |  |  |  |  |  |
| Heilongjiang | 33663 | | 1.66 | 31,850,088 | 1.057 | 106 | 3.149 | 11.0 (7.0 to 17.0) | 2.87 (1.25 to 4.47) |
| Jilin | 30098 | | 1.49 | 24,073,453 | 1.250 | 56 | 1.861 | 9.0 (5.0 to 16.0) | 3.07 (1.34 to 5.01) |
| Liaoning | 74804 | | 3.69 | 42,591,407 | 1.756 | 162 | 2.166 | 11.0 (6.0 to 19.0) | 2.47 (0.99 to 4.23) |
| **East China** |  | |  |  |  |  |  |  |  |
| Shanghai | 68875 | | 3.40 | 24,870,895 | 2.769 | 98 | 1.423 | 6.0 (4.0 to 10.0) | 4.22 (2.56 to 6.10) |
| Jiangsu | 181882 | | 8.98 | 84,748,016 | 2.146 | 248 | 1.364 | 10.0 (6.0 to 15.0) | 2.41 (0.98 to 3.95) |
| Zhejiang | 138134 | | 6.82 | 64,567,588 | 2.139 | 58 | 0.420 | 9.0 (6.0 to 14.0) | 1.95 (0.90 to 3.14) |
| Anhui | 91133 | | 4.50 | 61,027,171 | 1.493 | 107 | 1.174 | 10.0 (6.0 to 16.0) | 1.66 (0.68 to 2.87) |
| Fujian | 52851 | | 2.61 | 41,540,086 | 1.272 | 30 | 0.568 | 10.0 (6.0 to 15.0) | 2.51 (0.87 to 4.08) |
| Jiangxi | 54590 | | 2.70 | 45,188,635 | 1.208 | 70 | 1.282 | 11.0 (6.0 to 18.0) | 1.89 (0.69 to 3.39) |
| Shandong | 129614 | | 6.40 | 101,527,453 | 1.277 | 105 | 0.810 | 9.0 (5.0 to 14.0) | 2.05 (0.87 to 3.54) |
| **Central South China** |  | |  |  |  |  |  |  |  |
| Henan | 89906 | | 4.44 | 99,365,519 | 0.905 | 112 | 1.246 | 11.0 (6.0 to 18.0) | 1.73 (0.62 to 3.27) |
| Hubei | 97131 | | 4.80 | 57,752,557 | 1.682 | 109 | 1.122 | 12.0 (7.0 to 19.0) | 1.85 (0.66 to 3.35) |
| Hunan | 90336 | | 4.46 | 66,444,864 | 1.360 | 64 | 0.708 | 11.0 (6.0 to 19.0) | 1.37 (0.46 to 3.21) |
| Guangdong | 123785 | | 6.11 | 126,012,510 | 0.982 | 173 | 1.398 | 10.0 (5.0 to 18.0) | 2.20 (0.80 to 3.98) |
| Guangxi | 48422 | | 2.39 | 50,126,804 | 0.966 | 85 | 1.755 | 10.0 (5.0 to 17.0) | 1.65 (0.62 to 3.23) |
| Hainan | 11806 | | 0.58 | 10,081,232 | 1.171 | 10 | 0.847 | 11.0 (6.0 to 18.0) | 2.18 (0.71 to 3.95) |
| **Southwest China** |  | |  |  |  |  |  |  |  |
| Chongqing | 36111 | | 1.78 | 32,054,159 | 1.127 | 43 | 1.191 | 11.0 (6.0 to 19.0) | 1.71 (0.64 to 3.38) |
| Sichuan | 159258 | | 7.86 | 83,674,866 | 1.903 | 201 | 1.262 | 11.0 (6.0 to 18.0) | 1.49 (0.51 to 3.04) |
| Guizhou | 46204 | | 2.28 | 38,562,148 | 1.198 | 39 | 0.844 | 11.0 (6.0 to 18.0) | 1.35 (0.47 to 3.27) |
| Yunnan | 74806 | | 3.69 | 47,209,277 | 1.585 | 53 | 0.708 | 11.0 (6.0 to 17.0) | 1.51 (0.53 to 3.08) |
| Tibet | 3590 | | 0.18 | 3,648,100 | 0.984 | 1 | 0.279 | 10.0 (6.0 to 17.0) | 1.31 (0.55 to 3.25) |
| **Northwest China** |  | |  |  |  |  |  |  |  |
| Shaanxi | 59713 | | 2.95 | 39,528,999 | 1.511 | 45 | 0.754 | 9.0 (6.0 to 16.0) | 2.57 (1.16 to 3.97) |
| Gansu | 32084 | | 1.58 | 25,019,831 | 1.282 | 23 | 0.717 | 11.0 (7.0 to 17.0) | 1.40 (0.50 to 2.79) |
| Qinghai | 12592 | | 0.62 | 5,923,957 | 2.126 | 17 | 1.350 | 10.0 (6.0 to 15.0) | 1.57 (0.55 to 3.43) |
| Ningxia | 16107 | | 0.80 | 7,202,654 | 2.236 | 16 | 0.993 | 9.0 (6.0 to 15.0) | 2.09 (0.89 to 3.58) |
| Xinjiang | 33713 | | 1.66 | 25,852,345 | 1.304 | 60 | 1.780 | 10.0 (6.0 to 16.0) | 1.96 (0.75 to 3.51) |
| **Hong Kong** | | No Data | | | | | | | |
| **Macau** | | No Data | | | | | | | |
| **Taiwan** | | No Data | | | | | | | |
| **Total** | 2025169 | | 100.00 | 1,409,778,724 | 1.437 | 2448 | 1.209 | 10.0 (6.0 to 16.0) | 2.09 (0.77 to 3.77) |
