# Supplementary material for: Epidemiology and the economic burden of traumatic fractures in China: A population-based study
Source: Front Endocrinol (Lausanne). 2023 Jan 24;14:1104202. doi: 10.3389/fendo.2023.1104202 (PMC9902367; doi:10.3389/fendo.2023.1104202)
Supplement: Supplementary file 4 [file Table_4.docx]

**Supplementary table 4** The ten most common diagnoses in patients with traumatic fractures in China in 2020

| **Diagnose** | **Number** | **%** |
| --- | --- | --- |
| Femoral neck fracture | 138377 | 6.83% |
| Intertrochanteric fracture | 110898 | 5.48% |
| Clavicle fracture | 94824 | 4.68% |
| Rib fracture | 76025 | 3.75% |
| Lumbar vertebrae fracture | 65689 | 3.24% |
| Patella fracture | 61048 | 3.01% |
| Distal radius fracture | 48880 | 2.41% |
| Ankle fracture | 45762 | 2.26% |
| Humeral fracture | 42550 | 2.10% |
| Tibial fracture | 37981 | 1.88% |
