# Supplementary material for: Epidemiology and the economic burden of traumatic fractures in China: A population-based study
Source: Front Endocrinol (Lausanne). 2023 Jan 24;14:1104202. doi: 10.3389/fendo.2023.1104202 (PMC9902367; doi:10.3389/fendo.2023.1104202)
Supplement: Supplementary file 5 [file Table_5.docx]

**Supplementary table 5** The twenty most common procedures in patients with traumatic fractures in China in 2020

| **Procedure** | **Number** | **%** |
| --- | --- | --- |
| CRIF with intramedullary nails for femoral fractures | 67015 | 4.45% |
| ORIF with plates for tibial fractures | 58514 | 3.88% |
| ORIF with plates for clavicle fractures | 55374 | 3.68% |
| Hemiarthroplasty | 52386 | 3.48% |
| ORIF with plates for radius fractures | 49368 | 3.28% |
| Percutaneous vertebroplasty | 47296 | 3.14% |
| ORIF with plates for humeral fractures | 44584 | 2.96% |
| Total hip arthroplasty | 40367 | 2.68% |
| ORIF with plates for ankle fractures | 35622 | 2.36% |
| ORIF for lumbar vertebrae fractures | 33554 | 2.23% |
| ORIF with pins for phalangeal fractures | 26120 | 1.73% |
| ORIF with intramedullary nails for femoral fractures | 25430 | 1.69% |
| ORIF with plates for femoral fractures | 25080 | 1.66% |
| ORIF for tibial fractures | 25044 | 1.66% |
| ORIF with plates for calcaneal fractures | 23653 | 1.57% |
| Tension-band wire internal fixation for patella fractures | 23573 | 1.56% |
| ORIF for clavicle fractures | 22268 | 1.48% |
| ORIF for humeral fractures | 20911 | 1.39% |
| Percutaneous kyphoplasty | 20824 | 1.38% |
| ORIF for femoral fractures | 19717 | 1.31% |
